# Greater liver PNPLA3 protein abundance in vivo and in vitro supports lower triglyceride accumulation in dairy cows

**DOI:** 10.1038/s41598-021-82233-0

**Published:** 2021-02-02

**Authors:** Ryan S. Pralle, Sophia J. Erb, Henry T. Holdorf, Heather M. White

**Affiliations:** 1 Department of Dairy Science, University of Wisconsin-Madison, Madison, 53706 USA; 2 Department of Dairy Science, University of Wisconsin-Madison, 1675 Observatory Drive, Rm 934B, Madison, WI 53706 USA

**Keywords:** Animal physiology, Fat metabolism

## Abstract

Fatty liver syndrome is a prevalent metabolic disorder in peripartum dairy cows that unfavorably impacts lactation performance and health. Patatin-like phospholipase domain-containing protein 3 (PNPLA3) is a lipase that plays a central role in human non-alcoholic fatty liver disease etiology but has received limited attention in bovine fatty liver research. Thus, we investigated the relationship between tissue PNPLA3 expression and liver triglyceride accumulation in vivo via a ketosis induction protocol in multiparous dairy cows peripartum, as well as in vitro via small interfering RNA knockdown of *PNPLA3* mRNA expression in bovine primary hepatocytes. Results demonstrated a negative association (*P* = 0.04) between liver PNPLA3 protein abundance and liver triglyceride content in peripartum dairy cows, while adipose PNPLA3 protein abundance was not associated with liver triglyceride content or blood fatty acid concentration. Knockdown of *PNPLA3* mRNA resulted in reduced PNPLA3 protein abundance (*P* < 0.01) and greater liver triglyceride content (*P* < 0.01). Together, these results suggest greater liver PNPLA3 protein abundance may directly limit liver triglyceride accumulation peripartum, potentially preventing bovine fatty liver or accelerating recovery from fatty liver syndrome.

## Introduction

Accumulation of liver triglycerides (TG) is a common feature of the periparturient period in dairy cattle due to the negative energy balance and insulin resistance associated with the initiation of lactation^1–3^. Excessive accumulation of liver TG can result in clinical fatty liver syndrome (FLS), which has been associated with increased risk for other early lactation metabolic disorders, diseases, and impaired lactation performance^3^. Although there are inconsistencies with unit of measure (wet, dry, or DNA basis) reported, and few epidemiological studies to guide, proposed categories for FLS are: severe > 10%, moderate 5–10%, mild 1–5%, and normal < 1% liver TG, % wet weight basis^3^. With an estimated 50% of dairy cows experiencing some liver TG accumulation^4,5^, understanding the etiology of bovine FLS and developing liver TG mitigation strategies are of relevant interest to dairy production systems. A surprising gap in our knowledge is the role of liver lipases to the onset, progression, and recovery of bovine FLS^6^.

Patatin-like phospholipase domain-containing 3 (PNPLA3) is a lipase of interest in human medical research because of a genetic mutation (rs738409) associated with nonalcoholic fatty liver disease (NAFLD) and steatohepatitis^7–9^. Although PNPLA3 has some capacity for acyltransferase activity^10,11^, its predominate enzymatic activity is lipolysis^11,12^. Liver PNPLA3 has been proposed as a lipid droplet regulator in fed states by remodeling the lipid droplet FA profile^13,14^. There has been little investigation into the expression, regulation, and role of PNPLA3 in the etiology of dairy cow lipid-related metabolic disorders. Characterization of liver *PNPLA3* mRNA expression in dairy cows exhibited decreased expression at the time of parturition and during 50% feed restriction^15^. This observation of decreased expression during natural and induced negative energy balance parallels responsiveness to fasting in mice^12,16,17^ and suggests that the respective protein may also play a role in liver TG accumulation in dairy cows. Adipose *PNPLA3* expression has not been strongly linked to the pathology of human NAFLD, but is responsive to nutritional status^18,19^, glucose^20,21^, and energy status related hormones^20–22^. The dynamics of PNPLA3 protein abundance in the liver and adipose tissues of periparturient dairy cattle are still unknown.

We hypothesized that PNPLA3 protein maintains liver hydrolysis of TG and that liver downregulation at parturition permits accumulation of liver TG. Additionally, we hypothesized that PNPLA3 is present in bovine adipose tissue and would be greater during adipose TG mobilization. To evaluate these hypotheses, we conducted an in vivo experiment (Experiment 1) evaluating tissue PNPLA3 in multiparous cows subjected to either a control (CTL) or ketosis induction protocol (KIP) treatment, as well as an in vitro experiment (Experiment 2) implementing small interfering RNA (siRNA) knockdown of *PNPLA3* mRNA in bovine primary hepatocytes. The objectives of this complete work were to (1) elucidate liver and adipose PNPLA3 protein abundance dynamics across the peripartum period in multiparous dairy cows subjected to a ketosis induction protocol, (2) explore the potential in vivo association between tissue PNPLA3 and liver TG content peripartum, and (3) determine the direct impact of in vitro PNPLA3 knockdown on cellular TG content.

## Results

### Experiment 1: in vivo ketosis induction study

#### Cow biometrics and feed intake

**Figure 1:**
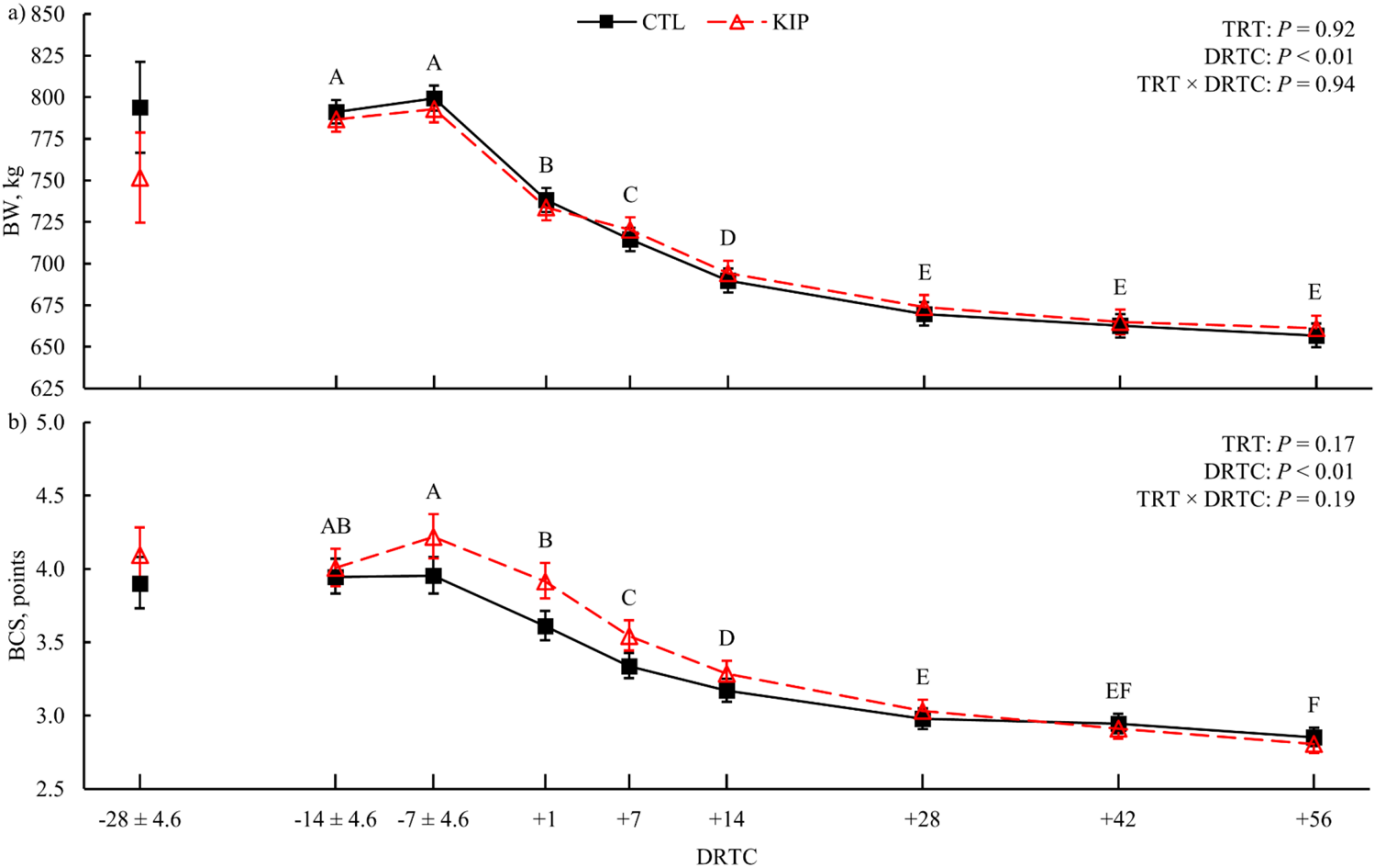
Body weight (BW) and body condition score (BCS) across the experimental period for control (CTL) and ketosis induction protocol (KIP) treatments (TRT). Error bars represent the 68% confidence limits of the least squares mean. The − 28 expected days relative to calving (DRTC) values were included as model covariates for their respective response; values graphically represented for − 28 expected DRTC are the arithmetic means. Variance in the actual DRTC sampled is represented for the relevant prepartum timepoints (± standard deviation). Differences across DRTC (*P* ≤ 0.05, Tukey's adjustment) are indicated when alphabetic superscripts do not share characters.

**Figure 2:**
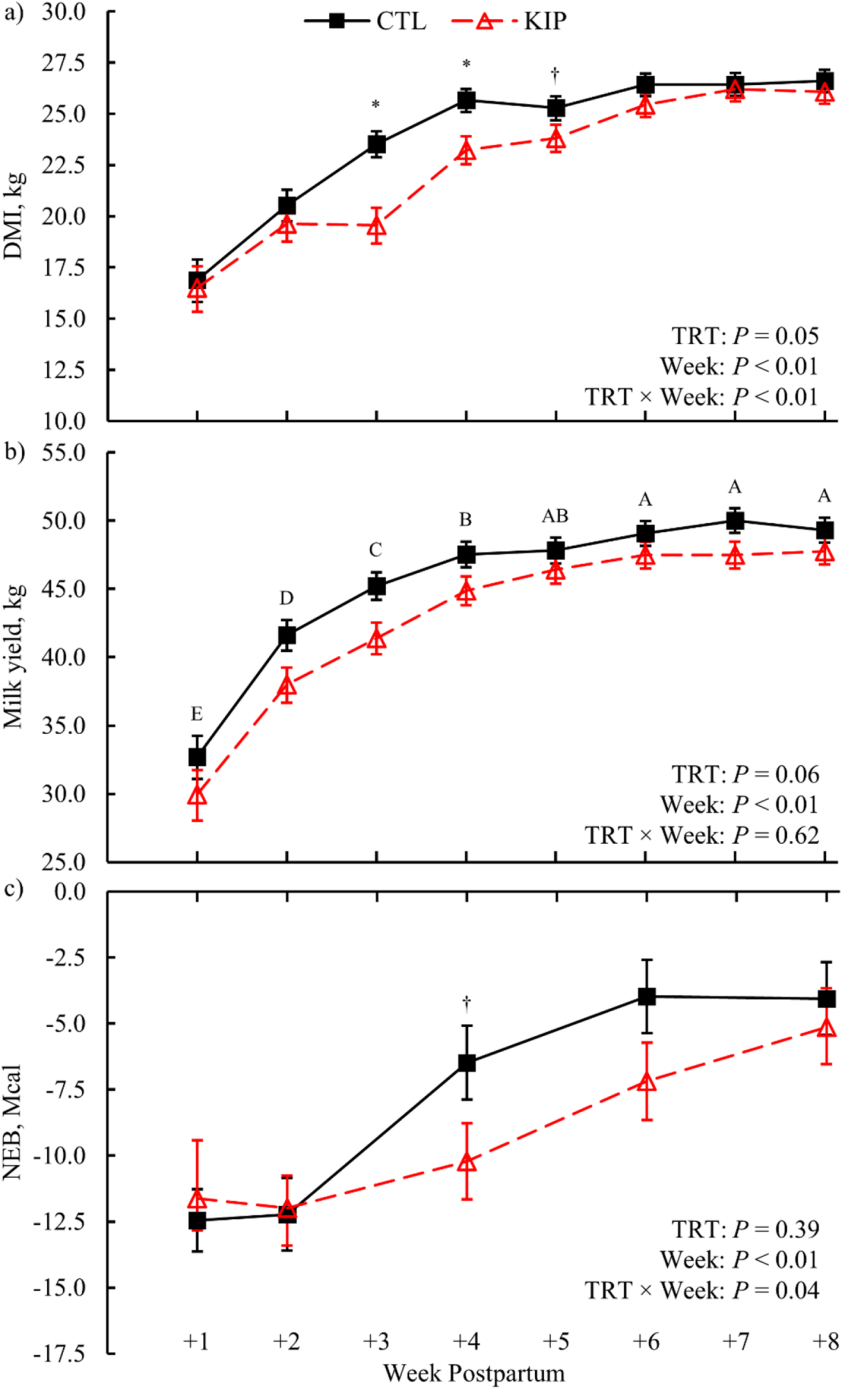
Weekly average dry matter intake (DMI; **a**), milk yield (**b**), and calculated net energy balance (NEB; **c**) during the postpartum period for control (CTL) and ketosis induction protocol (KIP) treatments (TRT). Error bars represent the 68% confidence limits of the least squares mean. Differences across weeks postpartum (*P* ≤ 0.05, Tukey's adjustment) are indicated when alphabetic superscripts do not share characters. Symbols represent significant (*P ≤ 0.05) and marginal (^†^0.05 < *P* ≤ 0.10) simple effect differences for treatment × time interactions after multiplicity correction (Bonferroni).

There was no evidence for treatment differences in body weight (BW; *P* = 0.92), body condition score (BCS; *P* = 0.17), prepartum BW change (*P* = 0.93), and postpartum change in BW (*P* = 0.63; Table 1). However, BW and BCS differed across the experimental period (*P* < 0.01; Fig. 1). Prepartum BCS loss was lower (*P* = 0.01) for KIP cows than CTL, but postpartum BCS loss was greater (*P* = 0.03) for KIP cows than CTL cows (Table 1). Additionally, birth weight of calves did not differ (*P* = 0.45) between treatments. Prepartum dry matter intake (DMI) and NE_L_ intake was greater (*P* = 0.01 and *P* < 0.01, respectively) for KIP cows than CTL cows; DMI and NE_L_ intake differed similarly across time (*P* < 0.01; Fig. 2a). Additionally, significant (*P* < 0.01) treatment × time effects were observed for postpartum DMI (Fig. 2a) and NE_L_ intake with significant (*P* < 0.01) simple effects detected at + 3 and + 4 weeks postpartum for both variables at which lower DMI and NE_L_ intake was observed for KIP cows than CTL cows. Refused feed as a % of feed offered had marginal evidence (*P* = 0.08) for a treatment × time effect, where KIP cows had greater % refusals from − 5 to − 3 weeks from parturition than CTL cows (*P* = 0.02, simple effects for all weeks). There was marginal evidence (*P* = 0.09, simple effect) on week 3 postpartum for KIP cows to have a lower % refusals than CTL cows. During feed restriction, only 2 KIP cows had feed refusals > 0%, 2 days each (Cow A: 1.2% and 1.2%; Cow B: 1.6% and 6.3%).

**Table 1:** Least squares means (LSM) and 95% confidence intervals (CI) of body weight, body condition score, dry matter intake, milk and milk component yield, milk composition, and energy balance for cows exposed to a control (CTL) or ketosis induction protocol (KIP) treatment.

Response^a^	CTL		KIP		*P*-value^b^		
	LSM	95% CI	LSM	95% CI	Trt	Time	T × T
BW, kg	715.4	[705.6, 725.0]	716.0	[705.9, 726.2]	0.92	< 0.01	0.94
**ΔBW, kg**							
Prepartum	2.9	[− 7.5, 13.3]	3.5	[− 7.3, 14.4]	0.93		
Postpartum	− 75.0	[− 90.6, − 59.6]	− 80.4	[− 96.5, − 64.2]	0.63		
Calf BW, kg	41.95	[38.73, 45.16]	40.85	[37.62, 44.08]	0.45		
BCS, pts	3.30	[3.17, 3.44]	3.39	[3.25, 3.54]	0.17	< 0.01	0.19
**ΔBCS, pts**							
Prepartum	− 0.33	[− 0.49, − 0.17]	− 0.01	[− 0.18, 0.15]	0.01		
Postpartum	− 0.82	[− 1.00, − 0.63]	− 1.13	[− 1.33, − 0.94]	0.03		
**DMI, kg**							
Prepartum	14.3	[12.7, 15.7]	15.9	[14.5, 17.1]	0.01	< 0.01	0.78
Postpartum	24.2	[23.2, 25.3]	22.9	[21.7, 24.0]	0.05	< 0.01	< 0.01
Feed refused, %	8.0	[6.36, 9.93]	9.87	[7.97, 11.98]	0.02	< 0.01	0.08
**NE**_**L**_** intake, Mcal**							
Prepartum	20.3	[17.9, 22.4]	25	[23.2, 26.6]	< 0.01	< 0.01	0.81
Postpartum	39.7	[37.8, 41.5]	37.4	[35.4, 39.4]	0.05	< 0.01	< 0.01
**Milk yield**							
Total, kg	45.8	[44.1, 47.4]	43.4	[41.5, 45.2]	0.06	< 0.01	0.62
Fat, kg	2.2	[2.0, 2.3]	2.2	[2.0, 2.3]	0.96	< 0.01	0.08
Protein, kg	1.3	[1.2, 1.4]	1.2	[1.1, 1.3]	0.19	< 0.01	0.98
Lactose, kg	2.2	[2.2, 2.3]	2.2	[2.1, 2.2]	0.16	< 0.01	0.76
Energy, Mcal	36.3	[34.0, 38.6]	35.7	[33.2, 38]	0.65	< 0.01	0.10
**Milk composition**							
Fat, %	4.66	[4.32, 5.07]	4.92	[4.55, 5.37]	0.25	< 0.01	0.81
Protein, %	2.83	[2.75, 2.92]	2.83	[2.75, 2.92]	0.97	< 0.01	0.04
Lactose, %	4.88	[4.80, 4.95]	4.94	[4.86, 5.01]	0.19	< 0.01	0.53
SnF, %	8.75	[8.61, 8.90]	8.81	[8.67, 8.96]	0.51	< 0.01	0.15
MUN, mg/dL	11.29	[10.82, 11.80]	11.26	[10.77, 11.79]	0.92	< 0.01	0.78
SCC × 1000	70.06	[32.43, 150.04]	61.99	[27.73, 137.10]	0.82	< 0.01	0.16
**EBAL, Mcal**							
Prepartum	7.9	[5.1, 10.3]	12.6	[10.4, 14.6]	< 0.01	0.04	0.27
Postpartum	− 7.8	[− 10.1, − 5.5]	− 9.2	[− 11.6, − 6.8]	0.39	< 0.01	0.04

^a^Body weight = BW, BW change = ΔBW, body condition score = BCS, prepartum changes are from − 28 to + 1 expected days relative to calving (DRTC) and postpartum are from + 1 to + 56 DRTC, solids not fat = SnF, milk urea nitrogen = MUN, somatic cell count (SCC; cells/mL × 1000); net energy of lactation (NE_L_) intake, milk energy and energy balance (EBAL) were based on published equations (NRC, 2001).

^b^Statistics for fixed effects are treatment (TRT), time (week), and the interaction of TRT and time (T × T).

#### Lactation performance and energy balance

Marginal evidence (*P* = 0.06) was found for KIP cows to yield less milk than CTL cows: 43.4 [41.5, 45.2] kg vs. 45.8 [44.1, 47.4] kg, respectively (Fig. 2b). Total milk yield, milk protein yield, and milk lactose yield were affected by time (*P* < 0.01), increasing in quantity until 6 weeks postpartum for both treatments. There was no evidence for treatment differences in milk protein (*P* = 0.19) or lactose yield (*P* = 0.17; Table 1). Milk fat yield had marginal evidence (*P* = 0.08) for a treatment × time difference with a marginal simple effect (*P* = 0.08) at + 5 weeks postpartum whereas KIP cows yielded more milk fat than CTL cows (2.4 [2.2, 2.6] kg for KIP cows vs. 2.2 [1.9, 2.4] kg for CTL cows). Milk energy yield had marginal evidence for a treatment × time difference (*P* = 0.10), but there was a lack of evidence for any simple effects (*P* ≥ 0.13). Most milk composition variables—fat, lactose, solids not fat, milk urea nitrogen, and somatic cell count (SCC)—had no treatment differences (*P* ≥ 0.19; Table 1), but were altered (*P* < 0.01) across time, generally decreasing in content as milk volume increased over time. Milk protein composition had a treatment × time difference (*P* = 0.04), where marginal evidence (*P* = 0.08) was observed at + 3 postpartum for greater protein content in KIP than CTL, 2.93 [2.82, 3.05] % protein and 2.80 [2.71, 2.90] % protein, respectively. Prepartum energy balance was greater (*P* < 0.01) in KIP cows than CTL (Table 1) and was affected by time (*P* = 0.04). Postpartum energy balance had a treatment × time difference (*P* = 0.04; Fig. 2c) with marginal evidence (*P* = 0.07) for a simple effect at + 4 weeks postpartum with a more negative energy balance for KIP cows than CTL cows, − 10.2 [− 13.2, − 7.2] Mcal and − 6.5 [− 9.3, − 3.6] Mcal, respectively.

#### Blood fraction and liver metabolites

**Figure 3:**
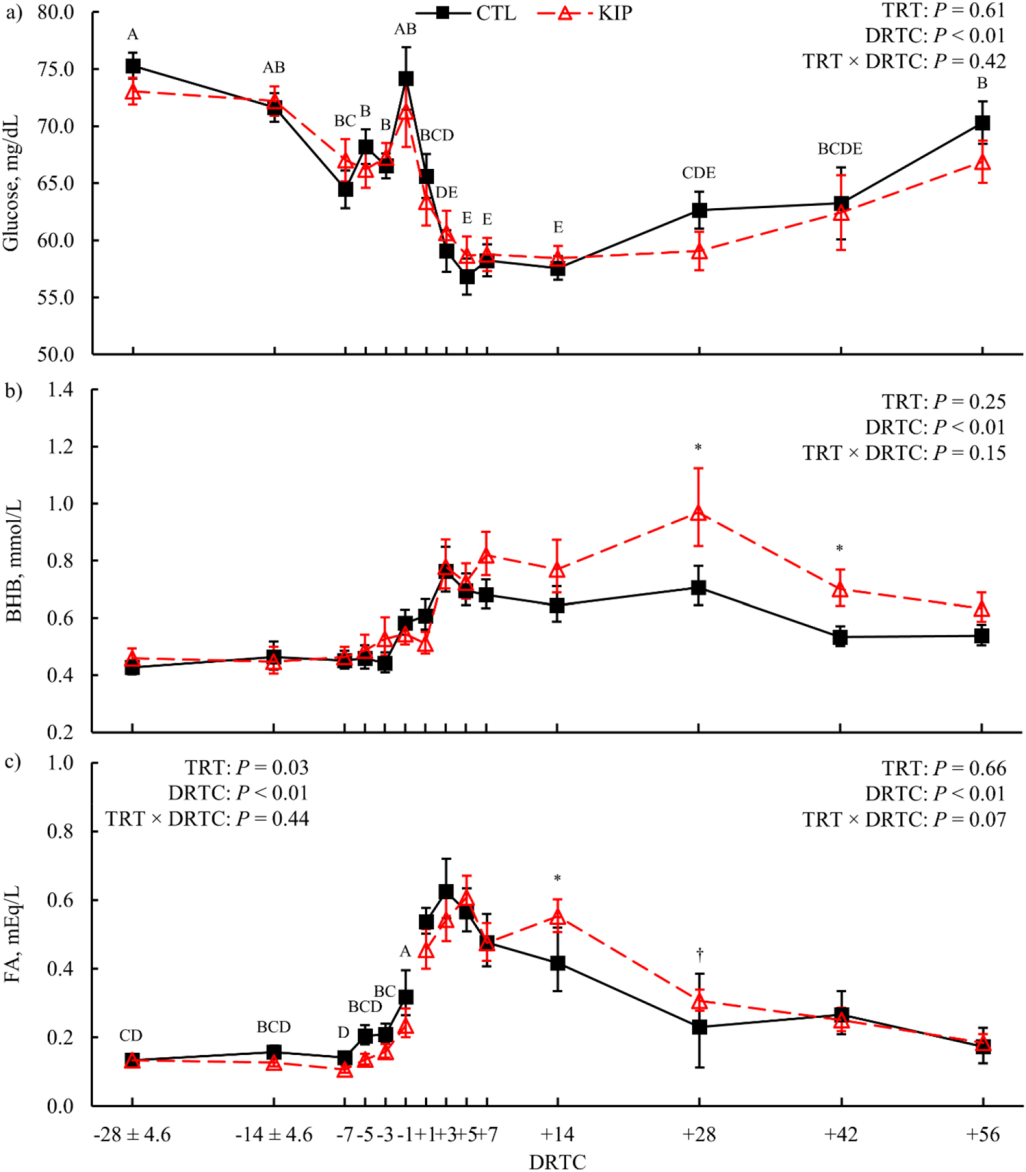
Plasma glucose (**a**), serum β-hydroxybutyrate (BHB; **b**), and plasma fatty acids (FA; **c**) during the experimental period for control (CTL) and ketosis induction protocol (KIP) treatments (TRT). Variance in the actual DRTC sampled is represented for the relevant prepartum timepoints (± standard deviation). Error bars represent the 68% confidence limits of the least squares mean. Differences across days relative to calving (DRTC, *P* ≤ 0.05, Tukey's adjustment) are indicated when alphabetic superscripts do not share characters. Symbols represent significant (*P ≤ 0.05) and marginal (^†^0.05 < *P* ≤ 0.10) simple effect differences for treatment by time interactions after multiplicity correction (Bonferroni).

**Figure 4:**
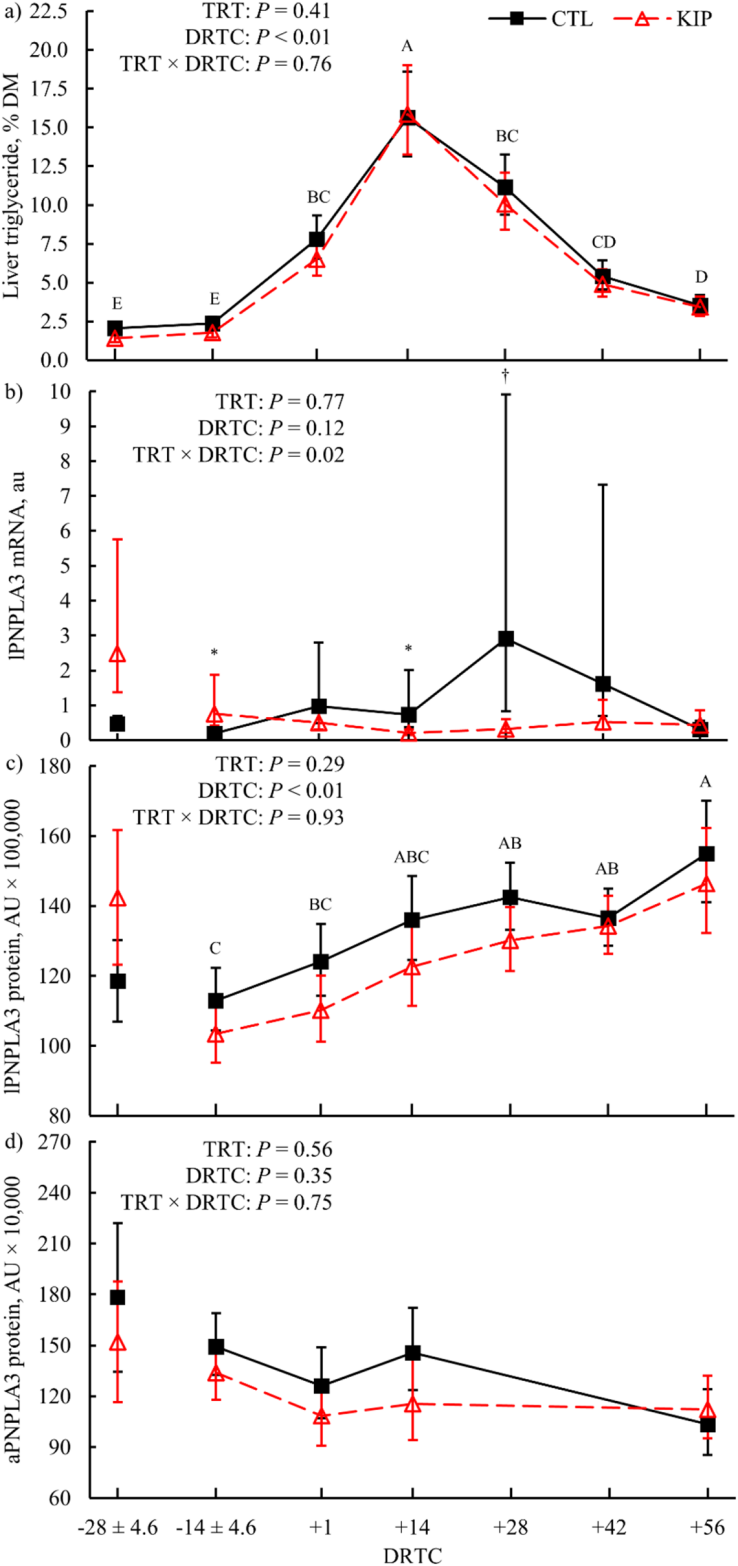
Liver triglyceride (**a**), liver patatin-like phospholipase domain-containing protein 3 (lPNPLA3) mRNA expression (**b**), lPNPLA3 protein abundance (**c**), and adipose PNPLA3 (aPNPLA3; **d**) protein abundance during the experimental period for control (CTL) and ketosis induction protocol (KIP) treatments (TRT). The − 28 expected days relative to calving (DRTC) values were included as model covariates for their respective responses (**b**–**d**); values graphically represented for − 28 expected DRTC are the arithmetic means. Variance in the actual DRTC sampled is represented for the relevant prepartum timepoints (± standard deviation). Error bars represent the 68% confidence limits of the least squares or arithmetic mean. Symbols represent significant (*P ≤ 0.05) and marginal (^†^0.05 < *P* ≤ 0.10) simple effect differences for treatment × time interactions after multiplicity correction (Bonferroni). Differences across expected days relative to calving (DRTC; *P* ≤ 0.05, Tukey's adjustment) are indicated when alphabetic superscripts do not share characters.

Plasma glucose was not affected by treatment (*P* = 0.61; Table 2) but did differ over time (*P* < 0.01) with concentrations from + 3 to + 14 days relative to calving (**DRTC**) being lower than prepartum and later postpartum timepoints (Fig. 3a). Serum β-hydroxybutyrate (**BHB**) had evidence (*P* = 0.15) for a treatment × time effect where KIP cows had greater BHB concentrations than CTL at + 28 (*P* = 0.05; Fig. 3b) and + 42 DRTC (*P* = 0.01; Fig. 3b). All KIP cows (n = 12) achieved the ketosis induction success threshold of blood BHB ≥ 3.0 mmol/L, as well as 2 CTL cows (3.2 ± 0.1 mmol/L, average ± SEM for both treatments). The average (± SD) and median DRTC cows achieved BHB ≥ 3.0 mmol/L was 17 (± 5) and 16.5 DRTC, respectively. Prepartum plasma fatty acid (FA) concentration was lower for KIP cows than CTL cows (*P* = 0.03; Table 2) and increased in concentration as parturition approached (*P* < 0.01; Fig. 3c). Postpartum plasma FA had marginal evidence (*P* = 0.07) for treatment × time differences in which KIP cows had greater FA than CTL cows on + 14 (*P* = 0.04; Fig. 3c) and + 28 DRTC (*P* = 0.07; Fig. 3c). No effect of treatment was observed for prepartum (*P* = 0.68) or postpartum serum TG concentrations (*P* = 0.23; Table 2). For both treatments, postpartum serum TG did change over time (*P* = 0.04; Table 2). Liver TG content was not impacted by treatment (*P* = 0.41, Table 2), but did differ over time (*P* < 0.01; Fig. 4a).

**Table 2:** Least squares means (LSM) and 95% confidence intervals (CI) of blood fraction metabolites, liver triglyceride (TG) content, and tissue patatin-like phospholipase domain-containing protein 3 (PNPLA3) expression for cows exposed to a control (CTL) or ketosis induction protocol (KIP) treatment.

Response^a^	CTL		KIP		*P*-value^b^		
	LSM	95% CI	LSM	95% CI	Trt	DRTC	T × D
Plasma glucose, mg/dL	65.27	[63.65, 66.89]	64.67	[62.97, 66.37]	0.61	< 0.01	0.42
Serum BHB, mmol/L	0.55	[0.51, 0.60]	0.59	[0.54, 0.66]	0.25	< 0.01	0.15
**Plasma FA, mEq/L**							
Prepartum	0.18	[0.15, 0.23]	0.14	[0.12, 0.17]	0.03	< 0.01	0.44
Postpartum	0.38	[0.32, 0.46]	0.40	[0.33, 0.48]	0.66	< 0.01	0.07
Liver TG, % DM	5.35	[4.12, 6.95]	4.59	[3.50, 6.03]	0.41	< 0.01	0.75
Liver PNPLA3 mRNA, au^c^	0.62	[0.27, 2.65]	0.41	[0.20, 1.23]	0.77	0.12	0.02
**PNPLA3 protein**^**d**^							
Liver, au × 100,000	133.84	[120.78, 148.32]	123.68	[111.07, 137.75]	0.29	< 0.01	0.93
Adipose, au × 10,000	129.84	[99.45, 169.51]	117.17	[89.45, 153.50]	0.55	0.34	0.78

^a^β-hydroxybutyrate = BHB, FA = fatty acid, prepartum analysis included samples collected from − 28 to 0 expected days relative to calving, postpartum analysis included samples collected from + 1 to + 56 days relative to calving.

^b^Statistics for fixed effects are treatment (TRT), day relative to calving (DRTC), and the interaction of TRT and DRTC (T × D).

^c^Standard error of the mean for the transformed (1/x^0.5^) *PNPLA3* mRNA relative expression data was 0.29 and 0.28 for CTL and KIP cows, respectively.

^d^Standard error of the mean Log_10_-transformed PNPLA3 protein abundance was 0.02 and 0.05 for liver and adipose tissue, respectively.

#### PNPLA3

**Figure 5:**
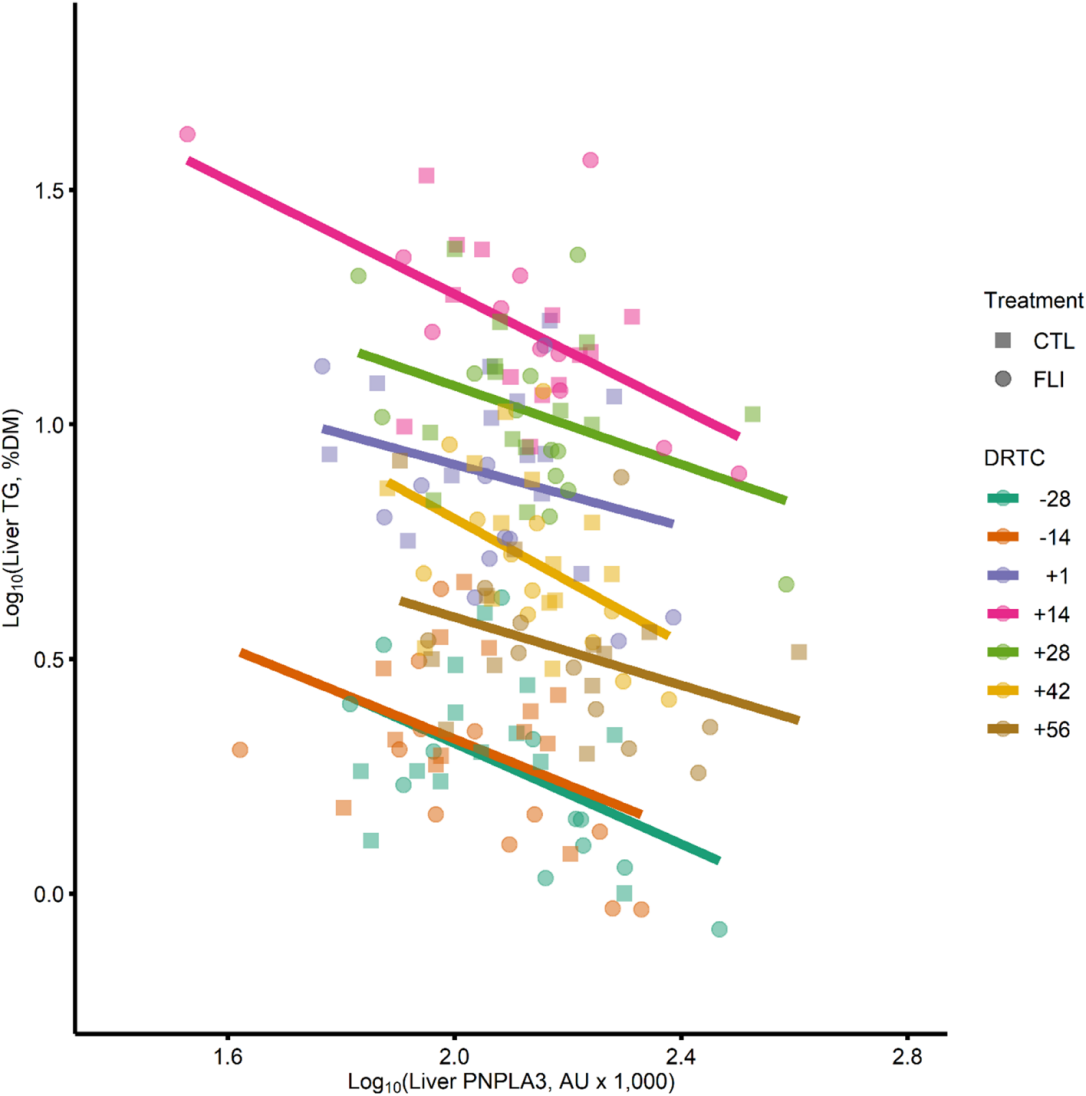
A representative plot for mixed effect regression analysis associating liver patatin-like phospholipase domain-containing protein 3 (PNPLA3) to liver triglyceride (TG) content. Liver PNPLA3 was negatively associated (β = − 0.31; *P* = 0.03) with liver TG when controlling for experimental design factors, including treatment, days relative to calving (DRTC), the interaction of treatment and time, and block effects. Magnitude of liver PNPLA3 abundance effect on liver TG content is represented by trendlines for the linear predictor within DRTC.

Relative abundance of *PNPLA3* liver mRNA expression had a significant (*P* = 0.02) treatment × time interaction, where KIP cows exhibited greater *PNPLA3* mRNA expression at − 14 DRTC (*P* = 0.02) and lower *PNPLA3* mRNA expression at + 14 (*P* = 0.04) and + 28 DTRC (*P* = 0.06) than the CTL cows (Fig. 4b). Liver PNPLA3 protein abundance was not altered by treatment (*P* = 0.29; Table 2) but was altered over time (*P* < 0.01; Fig. 4c). Adipose PNPLA3 was not affected by treatment (*P* = 0.56; Table 2) or time (*P* = 0.35; Fig. 4d). Least-squares means and SEM across DRTC for the transformed tissue PNPLA3 expression data are provided in Supplemental Table 1. Spearman correlations between − 28 DRTC PNPLA3 protein abundance and PNPLA3 at other DRTC timepoints were significant (*P* ≤ 0.05) within both tissues, with liver correlations ranging from 0.62 to 0.77 and adipose correlations ranging from 0.40 to 0.55 (Supplemental Table 2). Liver PNPLA3 protein abundance was significantly correlated with liver TG content (*P* = 0.01; r = − 0.32), plasma FA concentration (*P* < 0.01; r = − 0.39), and energy balance (*P* = 0.03; r = 0.27) but not with other metabolites (Supplemental Table 3). Mixed effect regression analysis elucidated a significant association between liver TG and liver PNPLA3 abundance (*P* = 0.04; β = − 0.34; Fig. 5); the model coefficient of determination^23^ (R^2^_V_) = 0.84 and the liver PNPLA3 abundance partial R^2^_V_ = 0.15.

### Experiment 2: primary bovine hepatocyte responses to siRNA

**Figure 6:**
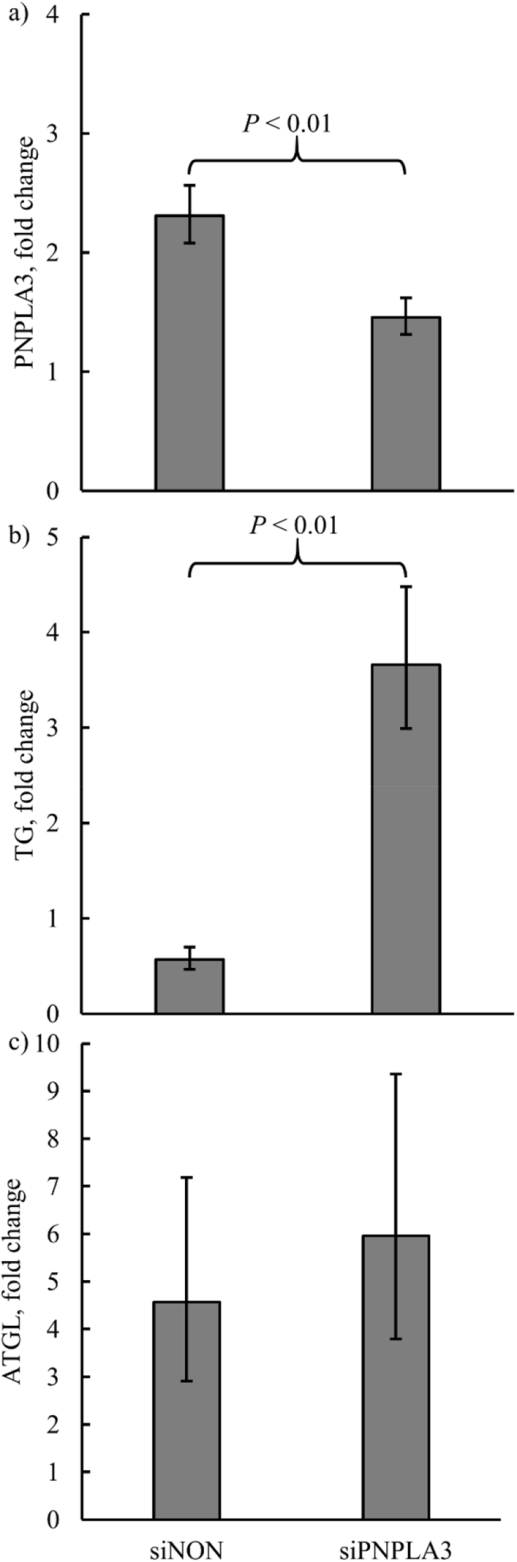
Fold changes in patatin-like phospholipase domain-containing protein 3 (PNPLA3) protein abundance (**a**), cellular triglyceride (TG; **b**) content, and adipose triglyceride lipase protein (ATGL) abundance (**c**) in cells transfected with a nonspecific small interfering RNA (siNON) or a PNPLA3 specific small interfering RNA (siPNPLA3) relative to basal media treated cells (double negative control). Error bars represent 68% confidence limits of the back-transformed least squares mean.

The in vitro data (protein abundance and cellular TG) were expressed relative to the basal media treated cells (i.e. cells not treated with any siRNA) and Log_2_ transformed. Location tests of the least squares means for PNPLA3 and adipose triglyceride lipase (ATGL) protein abundance were significantly different (*P* < 0.05) from 0 for both siRNA treatments, suggesting both treatments resulted in greater lipase protein abundance compared to basal media treated cells. The siPNPLA3 treatment had a lower (*P* < 0.01) positive PNPLA3 abundance fold change than siNON (Fig. 6a). Cellular TG was greater (*P* < 0.01) in response to siPNPLA3 compared to siNON (Fig. 6b). No difference in ATGL protein abundance (*P* = 0.64; Fig. 6c) was observed between siRNA treatments.

## Discussion

In the in vivo experiment, we examined the relationship between liver TG content and PNPLA3 expression in liver and adipose tissue, as well as the associations of PNPLA3 to energy metabolites over the transition to lactation period during a KIP protocol. The purpose of KIP was to exacerbate the physiological adaptation to lactation (e.g. negative energy balance, excessive mobilization of adipose TG) and generate a range of ketosis and fatty liver phenotypes in order to investigate the role of PNPLA3 in liver TG accumulation and recovery. Given the assumed parallel etiology of FLS and ketosis, and both the ease of using a cowside blood ketosis diagnostic and the inability to quantify liver TG content cowside or in real-time, ketosis was the marker of induction success.

By design, the KIP treatment had a greater prepartum dietary energy intake and positive energy balance prepartum due to the corn top-dress and modest increase in DMI. Concomitantly, plasma FA concentration was lower prepartum for KIP cows than CTL cows. These findings are consistent with previous experiments investigating the impact of dietary energy prepartum on cow performance^24–26^. Despite the greater prepartum energy intake, KIP cows did not have greater prepartum BW, BCS, or change in prepartum BW, which would be expected based on the aforementioned studies comparing different planes of prepartum dietary energy^24–26^. The lower prepartum BCS loss and plasma FA concentrations for the KIP cows compared to CTL cows suggested that the greater prepartum energy intake replaced energy mobilized from the subcutaneous adipose tissue mobilization. It is also possible that energy could have been partitioned to other tissues and the developing fetus. Calf birthweight did not differ; however, we did not measure calf tissue composition or fetal membranes.

The intentional reduction of postpartum DMI in KIP cows was verified statistically at + 3 weeks postpartum with KIP cows consuming 83.4% of CTL cow intake. Furthermore, KIP cows had significant evidence for lower DMI and NE_L_ intake than CTL cows at + 4 and + 5 weeks postpartum even after the re-alimentation of their diet to ad libitum, which coincided with greater negative energy balance, greater plasma FA concentration, and greater serum BHB concentration in KIP cows compared to CTL cows. The greater FA and BHB concentrations reflect the increased demand for endogenous energy stores to support lactation during the feed restriction period^1,2,27^, which is further supported by the greater BCS loss observed for the KIP cows. Both treatment groups had a relatively high milk fat %, which is an observation consistent with the relatively high BCS and BCS loss observed for both treatments^28,29^.

Our primary biomarker for the KIP protocol was blood BHB concentration measured cowside with 100% (n = 12) of KIP cows and 15% (n = 2) of CTL cows achieving BHB 3.0 ≥ mmol/L. Reaching this threshold resulted in cessation of feed restriction and treatment for ketosis. While results in the current study demonstrate a range of liver TG accumulation, the lack of treatment effect on liver TG content suggests that TG accumulation was not solely due to the KIP protocol. Fatty liver syndrome and ketosis are considered comorbid disorders with similar etiology^2^, and thus, we anticipated inducing ketosis would also result in an increase in liver lipids and although the KIP protocol did effectively induce ketosis cases (BHB ≥ 3.0 mmol/L) in KIP cows, the absence of a concomitant effort on liver TG suggests that either control cows were also under sufficient metabolic challenge to exhibit FLS or that FLS and ketosis do not always develop in parallel. Previous research using similar induction protocols^30–33^ also note that some cows have varying resistance to ketosis^32,34,35^ which could suggest a potential disconnect in etiology in a subset of cows. It is important to recognize that in the current experiment both treatment groups could be considered obese based on prepartum BCS^36^ and lost a substantial amount of BCS and BW postpartum. These factors have previously been associated with greater risk for ketosis and FLS^5,37–39^; therefore, it is possible that the CTL treatment were not representative of a preferred metabolic status. The observation of two CTL cows achieving the BHB ≥ 3.0 mmol/L absent of the KIP protocol further supported their inadequacy as a “healthy” metabolic control; thus, future experiments should consider limiting prepartum BCS prior to cow enrollment. Also, it may be important to account for susceptibility to metabolic disorder determined by prepartum metabolic characteristics or previous incidence of metabolic disorder^40,41^. Regardless, the observed lack of congruence between ketosis incidence and exacerbated liver TG content may suggest unique underlying physiology for both metabolic disorders.

The observed treatment × time differences in liver *PNPLA3* mRNA appeared to be concordant with the observed differences in NE_L_ intake, with KIP cows having greater mRNA and intake prepartum yet lower at + 14 and + 28 DRTC (or + 3 and + 4 weeks) than CTL. Fasting conditions in rodents result in a downregulation of *PNPLA3* mRNA expression and refeeding upregulates expression^42,43^ which was similarly observed in 50% feed restricted mid-lactation dairy cows^15^. This down regulation is thought to be mediated by metabolic factors, such as lower blood glucose, greater blood FA, and greater insulin^21,44^ and a negative correlation between *PNPLA3* mRNA expression and plasma FA was previously documented in dairy cows^15^. Despite the pattern of *PNPLA3* expression herein, we did not observe any significant associations between postpartum *PNPLA3* mRNA, calculated energy balance, or blood fraction metabolite concentrations indicative of energy status (*i.e.* glucose and FA). Liver PNPLA3 protein abundance was not affected by dietary treatment or treatment × time. This observation was not in agreement with the mRNA differences between KIP and CTL cows. The absence of the expected changes in liver PNPLA3 protein abundance, based on mRNA data, may be due to posttranslational regulation. Saturated (C16:0), monounsaturated (C18:1), and polyunsaturated (C18:2) FA can extend PNPLA3 protein half-life^44^. Considering the greater concentration of plasma FA at + 14 and + 28 DRTC for KIP cows compared to CTL, a greater efflux of the aforementioned regulatory FA from adipose tissue^45^ could have maintained liver PNPLA3 protein abundance by reducing the protein degradation rate. While this theory agrees with the present literature, liver PNPLA3 abundance was negatively correlated to plasma FA concentration and positively correlated to energy balance. Additionally, *PNPLA3* mRNA expression was not correlated to these variables. Together, these could indicate independent regulation by energy balance and FA on liver PNPLA3 abundance, likely at the translational or post-translational level. Overall, these results suggest a complex integration of regulatory mechanisms targeting liver *PNPLA3* mRNA expression and its resulting protein abundance that merit further interrogation.

Ultimately, a lack of treatment effect on PNPLA3 protein abundance may not be surprising given the lack of difference in average liver TG content between treatments. In order to examine the relationship between PNPLA3 protein abundance and liver TG on an individual cow basis, mixed effect regression analysis and partial Spearman correlations were employed and revealed that liver PNPLA3 protein abundance was negatively associated with liver TG content over the peripartum period and within each timepoint. Correlations only demonstrate associations and therefore the in vitro experiment was completed to determine a direct relationship between PNPLA3 abundance and TG accumulation in primary bovine hepatocytes. The siPNPLA3 treatment did successfully knockdown PNPLA3 protein abundance compared to siNON in primary hepatocytes, which was concomitant with greater cellular TG for the siPNPLA3 treated cells. To confirm the greater cellular TG was not the result of an untargeted knockdown of ATGL, a PNPLA3 paralog and the rate limiting lipase^46,47^, we probed for its protein abundance and found ATGL was unaffected by siRNA treatment. Thus, the in vivo and in vitro results together suggest liver PNPLA3 protein may have a direct mechanism contributing to the net retention of liver TG in peripartum dairy cows. Because in vivo liver and in vitro hepatocyte TG content are lower with greater PNPLA3 protein abundance, the intuitive mechanism bovine liver PNPLA3 contributed to TG accumulation was lipolysis versus acyltransferase activity^10–12^.

The precise regulation of adipose tissue lipolysis at parturition in dairy cows is not fully understood and the present investigation into adipose PNPLA3 is novel in dairy cows. Although accepted as the rate-limiting lipase in other species^47^, ATGL was not increased postpartum when lipolysis is greatest^48^ suggesting that additional lipases may play an important role. Given that PNPLA3 contributes to lipolysis and potentially lipogeneses as an acyltransferase^10,12,13^, it was of interest to explore it within this experiment. In the current study, adipose PNPLA3 protein abundance did not appear to be influenced by treatment or have a significant temporal pattern and there were no correlations with metabolites, energy status, or body composition observed for adipose PNPLA3 protein. Adipose *PNPLA3* mRNA responsiveness to energy status has been demonstrated in rodents^16,19^, but posttranslational regulation of adipose PNPLA3 has not been confirmed. Nonetheless, the present lack of any significant associations with metabolites or energy status make it difficult to suggest responsiveness of adipose PNPLA3 protein in dairy cows. It may be that PNPLA3 does not contribute to peripartum lipolysis; however, there are several factors that could have contributed to the lack of observed responsiveness to energy status. In mice, subcutaneous adipose depots had less *PNPLA3* mRNA expression compared to internal adipose depots and mice of relatively greater age (6 vs 3 months) had less basal PNPLA3 mRNA expression^19^. Therefore, it is possible that the lack of results for any PNPLA3 protein abundance differences may be due to our use of mature cows or sampling of subcutaneous adipose tissue. Additionally, adipose *PNPLA3* mRNA expression in mice with genetic-induced obesity and diet-induced obesity appeared to no longer respond to carbohydrate refeeding^18,19^. As mentioned, the cows within this experiment had BCS values indicating that they were relatively obese, which may have blunted adipose tissue PNPLA3 responses. We propose that future investigations into the contribution of adipose tissue PNPLA3 to lipolysis should consider sampling from other adipose depots, primi- and multiparous cows, as well as cows across a range of body condition.

## Conclusions

The present work provided several novel insights into tissue specific PNPLA3 protein abundance in peripartum, multiparous dairy cows. First, peripartum adipose PNPLA3 protein abundance remained constant from − 14 to + 56 DRTC, suggesting it was nonresponsive within our experimental conditions. Second, liver *PNPLA3* mRNA expression was modulated by a KIP protocol, but liver PNPLA3 protein abundance was not affected. This could be due to an integration of translational and posttranslational mechanisms related to energy balance and adipose tissue FA mobilization. Third, liver PNPLA3 had a significant, negative association with liver TG content across the peripartum period, which suggests its relevance in preventing liver TG accumulation and liver TG clearance postpartum. The accompanying in vitro experiment further substantiates that reductions in PNPLA3 protein abundance have an inverse mechanistic effect on liver TG content in dairy cows. Additional in vitro experiments may further delineate regulatory mechanisms that could be leveraged to promote *PNPLA3* expression and its resulting protein abundance to prevent onset of, or accelerate recovery from, bovine FLS.

## Materials and methods

All animal use and handling protocols were approved by the University of Wisconsin-Madison College of Agricultural and Life Sciences Animal Care and Use Committee (protocol A005467-R01) and in accordance with the relevant guidelines of the same. Additionally, this work complied with the essential ARRIVE guidelines recommended by the National Centre for the Replacement, Refinement, and Reduction of Animals in Research (London, UK).

### Experiment 1: experimental design

Pregnant, multiparous Holstein cows (n = 25) were enrolled in the experiment conducted at the University of Wisconsin-Madison Dairy Cattle Instruction and Research Center (Madison, WI) and housed individually in tie-stalls. Cows were blocked by expected calving date and randomly assigned to a CTL (n = 13) or KIP (n = 12) treatment. Enrollment began at − 28 expected DRTC. The CTL treatment was allowed ad libitum intake of rations formulated to meet the needs of dry or lactating cows, respectively (Supplemental Table 5). Cows within the KIP treatment were subjected to a protocol similar to previous work^30,40,49^. Our protocol for the KIP treatment was to offer 6 kg of dry, cracked corn as a daily top-dress to each cow in addition to their ad libitum access to the dry cow ration (Supplemental Table 5) until parturition. After parturition, KIP cows were offered ad libitum access to the lactating cow ration (Supplemental Table 5) until + 14 DRTC, at which time feed intake was restricted to 80% of previous ad libitum intake. The previous ad libitum intake of each individual cow was calculated as the respective cow’s average voluntarily feed intake for the 3 days prior to the feed restriction period. Feed restriction ended when a cow achieved a blood BHB ≥ 3.0 mmol/L. All KIP cows achieved this threshold and the median number of d feed restricted was 2 (range from 0 to 12 days). Postpartum, blood BHB was monitored daily cowside for all cows with a BHBCheck meter (PortaCheck, Moorestown, NJ). Any cows that achieved a blood BHB ≥ 3.0 mmol/L (n = 2 for CTL; n = 12 for KIP) were treated for ketosis with intravenous dextrose (250 mL; Phoenix Scientific Inc., St. Joseph, MO; 50% dextrose), orally administered Propylene Advantage (300 mL/day for 3–5 days; TechMix LLC, Stewart, MN), and a B-vitamin complex injected intramuscularly (20 ml; Sparhawk Laboratories, Inc., Lenexa, KS). In addition, KIP cows undergoing feed restriction were re-alimented to feed and allowed ad libitum intake for the remainder of the experiment.

### Experiment 1: sample collection and analysis

Daily feed offered and refused was recorded by trained herd staff for each individual cow and daily feed intakes were determined by calculation. Individual feed ingredients and total mixed ration samples were collected weekly. Feed ingredients were dried in a 55 °C forced-air oven for 48 h, ground through a 1 mm screen by a Wiley Mill (Thomas Scientific, Swedesboro, NJ), and equal mass of dry, ground feed were composited by month. Composited samples were analyzed for composition by a commercial laboratory (Dairyland Labs, Arcadia, WI). Daily intake of net energy of lactation (NE_L_) was calculated based on published equations employed in the NRC dairy model software^50^ using the chemical composition of composited feed ingredients. The maintenance NE_L_ requirement was calculated as described by the NRC^50^: 0.08 × BW^0.75^. Milk NE_L_ yield was calculated based on the following NRC equation^50^: [(0.0929 × % milk fat) + (0.0563 × % milk true protein/0.93) + (0.0395 × % milk lactose)] × milk yield. Pregnancy NE_L_ requirement was calculated based on the following NRC equation^50^: [(0.00318 × day of gestation − 0.0352) × (calf BW/45)]/0.218. Net energy balance was calculated using the weekly averages of NE_L_ requirements and intake with the following equation: NE_L_ intake − milk NE_L_ yield − maintenance NE_L_ − pregnancy NE_L_.

Milking occurred 2 × daily and milk yield was recorded at every milking (BouMatic Xcalibur Herringbone, BouMatic, Madison, WI). For composition analysis, composite milk samples were collected at 4 consecutive milkings each week, preserved with 2-bromo-2-nitropropane-1,3-diol, and refrigerated at 4 °C until shipping (within 1 day of last consecutive sample) for analysis. Milk composition of fat, protein, lactose, solids not fat, milk urea nitrogen, and SCC were determined by a DHIA laboratory (AgSource, Menominee, WI). All milk samples were preheated to 40 °C and mixed before analysis of milk fat and milk protein by FTIR using the Foss MilkoScan FT + (Foss Analytical, Hillerød, Denmark) in accordance with the instrument manufacturer’s instructions and ISO 9622/ IDF 141:2013 (AOAC official method 972.16; AOAC International, 2016). Analysis of SCC was performed using Fossomatic FC (Foss Analytical). Per the DHIA’s standard operating procedures, milk samples were analyzed on equipment that is calibrated weekly with 12 standards, and standards are rechecked daily and hourly with a subset of 6 of the 12 standards. Intra-assay coefficients of variation for all variables were maintained at < 7%. Inter-assay coefficients of variation are not available for all variables; however, inter-assay coefficients of variation for fat and protein are maintained at < 2% and < 1.5%, respectively.

Body weights, BCS, blood samples, and liver biopsies were collected at − 28, − 14, + 1, + 14, + 28, + 42, and + 56 DRTC and additional blood samples were taken at − 7, − 5, − 3, − 1, + 3, + 5, + 7 DRTC. Adipose biopsies were performed on − 28, − 14, + 1, + 14, and + 56 DRTC. The − 28 and − 14 DRTC samples were collected based on expected calving date; across all cows, calving occurred 0.28 ± 4.59 days (average ± SD) relative to due date. Prepartum samples from − 7 to − 1 DRTC were the product of every other day sampling beginning 7 days prior to each cow’s due date. Two trained individuals independently recorded BCS using a five-point scale^36^ and the scores were averaged within an observation. Blood samples were collected by venipuncture of the coccygeal vessels before feeding at approximately 0800 h into evacuated tubes with or without additive.

Serum was separated from blood collected in tubes without additive (BD Vacutainer, Franklin Lakes, NJ) after centrifuging at 2500×*g* for 15 min at room temperature. Plasma was separated from blood collected in an evacuated tube containing potassium oxalate and 4% sodium fluoride (BD Vacutainer, Franklin Lakes, NJ) by centrifuging at 2000×*g* for 15 min at 4 °C. Serum and plasma aliquots were stored at − 20 °C until metabolite analysis. Plasma glucose, serum BHB, and serum TG concentrations were quantified in their respective aliquots using Catachem VETSPEC reagents on the Catachem Well-T AutoAnalyzer (Catachem, Awareness Technologies, Oxford, CT). All standards were within expected, calibrated ranges provided by the manufacturer during the calibration event (Catachem, Oxford, CT). Samples were read by the autoanalyzer in cuvettes either in duplicate (plasma glucose, serum BHB) or triplicate (serum TG). Methods for glucose (C124-06, Catachem), BHB (C444-0A, Catachem), and TG (C116-0A, Catachem) are based on the work of León et al. (1977), Koch and Feidbruegge (1987), and Trinder (1969), respectively. A serial dilution (1:2) of a standard (NEFA Standard Solution, FUJIFILM Wako Diagnostics U.S.A., Mountain View, CA) was used to establish a standard curve for quantification of serum TG. Plasma FA concentration was quantified enzymatically using a plate adaptation of the Catachem assay (C514-0A)^51–53^. Aliquots of a reference pool sample, respective to each sample type, were utilized for assay quality control. Intra-assay coefficient of variation never exceeded 10% for the quantification of the preceding blood fraction and liver metabolites. Inter-assay coefficients of variation were 5.9%, 6.5%, 5.6%, and 15.3%, for plasma glucose, plasma FA, serum BHB, and serum TG, respectively.

Liver samples (~ 750 mg) were obtained by blind percutaneous biopsy utilizing a custom built trocar^54,55^. Adipose tissue samples (~ 1 g) were obtained by punch biopsy as explained in the Supplemental Methods. Tissue biopsy samples were immediately rinsed with saline, aliquoted into tubes (~ 250 mg liver per tube; ~ 500 mg adipose per tube), frozen in liquid nitrogen, and stored at − 80 °C until further analysis of liver TG and protein abundance. As described by Caputo Oliveira et al. (2020), liver TG content was quantified by colorimetric assay of Folch-extracted product and expressed as a % of dry matter^56–58^.

Detailed methods for liver RNA isolation has been previously described^58^. Briefly, RNA was extracted via the manufacturer protocol (15596018; Life Technologies, ThermoFisher Scientific, Rockford, IL) using a phenol–chloroform extraction^59^, purified, and subjected to on-column DNAse I (Bio-Rad Laboratories, Hercules, CA) digestion using the Aurum Total RNA 96 Kit (732-6800; Bio-Rad Laboratories)^60^. Total RNA was eluted and then quantified using a Synergy H1 Hybrid Spectrophotometer (BioTek, Winooski, VT) as described^58,61^. Ratio absorbance of 260/280 nm of total RNA was between 1.9 and 2.1 and further assessed using the Bioanalyzer 2100 (Agilent Technologies, Santa Clara, CA) and to obtain an RNA integrity number (RIN; mean ± SD was 6.69 ± 1.28). Quality-assured RNA (1 µg) was reverse transcribed to cDNA in a 20 µL reaction using iScript Reverse Transcription Supermix (170-8840; Bio-Rad Laboratories) in a C1000 Touch Thermo Cycler (Bio-Rad Laboratories) according to the manufacturer (Bio-Rad Laboratories)^58,61^. A cDNA pool was made from equal volumes of each sample and was serial diluted 1:4 to make a 5-point standard curve for gene expression analysis using real time qPCR (RT-qPCR). Individual cDNA samples were diluted 1:10 before RT-qPCR analysis. Gene expression was measured using RT-qPCR in a CFX-384 Real-Time System (Bio-Rad Laboratories) utilizing SsoAdvanced SYBR (172–5270, Bio-Rad Laboratories). Primers used—ribsomal subunit 18S (*18S*), ribosomal protein L32 (*RPL32*), *PNPLA3*—were validated previously or within this experiment (Supplemental Table 6) and optimized using a no template control (nucleotide-free water), no reverse-transcription control (RNA pool), and 1:4 serially diluted cDNA 5-point standard curve. Standards, controls, and samples were amplified in triplicate for *18S* and *RPL32* as follows: 1 cycle at 95 °C for 3 min, 45 cycles of 95 °C for 15 s and 55 °C for 5 s, then a melt curve from 65 to 95 °C at increasing increments of 0.5 °C for 3 s. Primers for *PNPLA3* used an annealing temperature of 55.3 °C instead of 55 °C (Supplemental Table 6). Abundance of *18S* and *RPL32* were interrogated for their stability independently and in combination (geometric mean) using NormFinder^62^. The geometric mean of *18S* and *RPL32* was more stable (*M* = 0.093) than *18S* (*M* = 0.472) or *RPL32* (*M* = 0.432) independently; therefore, the starting quantity of *PNPLA3* mRNA for each samples was expressed relative to its respective geometric mean of *18S* and *RPL32* starting quantities.

### Experiment 2: hepatocyte isolation, culture, and treatment

Primary bovine hepatocytes were isolated from 3 Holstein bull calves less than 7 days old (average ± SD; 5 ± 2 days) with each individual calf represented an individual biological replicate. Isolation and initial culturing of primary hepatocytes is detailed in the Supplemental Methods. Wells were randomly assigned to experimental treatment in triplicate: basal media, siNON, or siPNPLA3. Cells were treated in two parallel blocks: one block for cellular TG content quantification and the other block for PNPLA3 protein abundance. All cells were treated with sterile Opti-MEM (Gibco, ThermoFisher Scientific, Rockford, IL). Cells destined to siRNA treatments were treated with separate mixtures of each respective siRNA reconstituted in 1 × siRNA buffer (B-002000-UB-100, Dharmacon, Horizon Discovery, Cambridge, United Kingdom), Lipofectamine 2000 (Invitrogen, ThermoFisher Scientific, Rockford, IL), and Opti-MEM. The siPNPLA3 treatment delivered a *PNPLA3* specific siRNA sequence at 80 µmol/L while the siNON treatment delivered a nonspecific siRNA sequence at 20 µmol/L. Cells were treated with siRNA and incubated for 4 h before media was changed to sterile Dulbecco’s Modified Eagle’s Medium (D2902, Sigma-Aldrich, St. Louis, MO) with added cell culture grade HEPES and sodium bicarbonate (DMEM; Sigma-Aldrich) supplemented with 1% bovine serum albumin (fatty acid free probumin, 820024, MilliporeSigma, Burlington, MA) until harvest for each respective fate 24 h later as done previously (White et al., 2011).

### Experiment 2: sample analysis

In one block of parallel treatments, media was removed, cells rinsed with 1 mL Ca-free Krebs buffer (pH 7.4), and then harvested in 750 µL dissociation buffer (2.68 mmol/L KCl, 1.47 mM KH_2_PO_4_, 137 mM NaCl, 8.06 mM Na_2_HPO_4_, 1.0 mM Na_2_ EDTA-2H_2_O, pH 7.4) and stored at − 20 °C for subsequent cellular TG and DNA analysis. After thawing on ice, plates were rinsed with 750 µL ice-cold dissociation buffer, cells were transferred to RNA-free tubes then sonicated and aliquoted for either TG or DNA analysis. An aliquot was used for total lipid extraction using a previous method^56^ with re-suspension of lipids in 300 µL of 1% Triton X-100 in 100% chloroform. Concentration of TG was determined in triplicate via colorimetric assay (L-Type Triglyceride M, Wako Diagnostics, Richmond, VA) using modified volumes of sample (10 µL) and reagents (R1: 90 µL; R2: 30 µL) of the manufacturer’s protocol (Wako Diagnostics). All samples fell within the multi-lipid calibrator 5-point standard curve diluted 1:2 and intra- and inter- assay CV never exceed 10%. The second aliquot was used for DNA quantification using the PicoGreen method (P7589; Molecular Probes, Thermo Fisher) via the manufacture’s protocol (Thermo Fisher) using 25 mM Tris–HCl, 20 mM EDTA buffer (pH 7.5) to dilute the reagent as previously described^63^. A 5-point standard curve of calf thymus DNA at a 1:2 dilution was assayed with samples all in triplicate^61^. All samples fell within the standard curve were assayed in triplicate and intra- and inter- assay CV never exceed 10%. Sample cellular TG content was normalized by dividing TG content by DNA content within each sample.

### Semi-quantitative western blotting

Semi-quantitative western blotting was used to analyze the abundance of PNPLA3 in adipose, liver tissue, and primary hepatocytes. Detailed sample preparation and Western methods are provided in the Supplemental Methods. Due to apparent differences in relative PNPLA3 abundance between tissues, the primary PNPLA3 antibody (ab81874; Abcam, Cambridge, MA) was diluted to 1:250 for adipose tissue and 1:500 liver tissue and primary bovine hepatocyte samples for a 1 h room temperature incubation. After primary incubation, blots were washed and the secondary antibody (diluted 1:5000 for all samples types; ab97080, Abcam) and HRP conjugate (161-0381; Bio-Rad Laboratories, Richmond, CA) were applied for 1 h at room temperature. Blot images for total lane protein and the PNPLA3 band were captured on the ChemiDoc XRS + Imager using Image Lab 5.0 software (Bio-Rad Laboratories, Richmond, CA). The abundance of PNPLA3 protein was normalized to total lane protein within Image Lab 5.0 (Bio-Rad Laboratories, Richmond, CA) for statistical evaluation. Hepatocyte blots were also probed for ATGL abundance with the procedure detailed above, except the primary antibody (ab99532; Abcam) diluted to 1:3000 was incubated overnight at 4 °C and a different secondary antibody (ab97051, Abcam) diluted 1:5000 was used (Supplemental Methods).

### Statistical analysis

Data analysis was performed using the SAS software (version 9.4; copyright 2002 to 2012, SAS Institute Inc., Cary, NC) procedures UNIVARIATE, CORR, and GLIMMIX. For response variables in both experiments, several distributions were found to have non-Gaussian distributions based on the Shapiro–Wilk test (*P* < 0.05). In those instances, the response variable data was systematically evaluated for transformations that provided Gaussian distributions empirically by Shapiro-Wilks test (*P* > 0.05) or subjectively by histogram visualization (when empirical solutions were not found). Dry matter intake, NE_L_ intake, serum TG, plasma FA, and calculated net energy balance had bimodal distributions due to different pre- and postpartum distributions; therefore, the data for these responses were subset by parturition status for further statistical analysis. Selected transformations for each response variable in experiment 1 are provided in Supplemental Table 4. Response variable expression and transformations for experiment 2 are detailed subsequently.

#### Experiment 1

Linear mixed models (LMM) were used to evaluate evidence for differential effects for all response variables. The fixed effects included treatment, time, and treatment × time; the random effects included cow, block (expected calving week), cow nested within week of lactation (models with subsampling), and repeated measures of cow across time. A systematic procedure was implemented for the development of the LMM for each response (Supplemental Methods; Supplemental Table 4). We considered fixed effects with *P* ≤ 0.05 as having significant evidence for differences and effects with 0.05 < *P* ≤ 0.10 as having marginal evidence for differences^64^. Whenever a treatment × time effect *P* ≤ 0.15 we made simple-effect comparisons between treatments within timepoint and corrected for multiplicity by the Bonferroni method^49,64^. A less conservative screening threshold (*P* ≤ 0.15) determined a priori was used for testing interaction simple effects because interactions are notoriously under power^64–67^. Treatment means are expressed as least squares means and their respective 95% confidence interval is denoted as [lower limit, upper limit] in text.

Preliminary associations between PNPLA3 (mRNA expression or protein abundance) and other variables of interest were performed on postpartum samples (values not transformed) via partial Spearman correlations controlling for treatment. Based on the significant partial correlation between liver PNPLA3 protein abundance and liver TG content, a LMM was fitted to better delineate the association between liver PNPLA3 protein abundance and liver TG content while accounting for experimental design and data structure. The mixed effect regression model was developed by fitting an initial model using final LMM model for liver TG (Supplemental Table 4) with liver PNPLA3 abundance as a fixed effect. Inclusion of higher order interactions between liver PNPLA3 and the other fixed effects (i.e. treatment or DRTC) did not minimize BIC, provide evidence for effects (*P* ≤ 0.15), or improve residuals. Multicollinearity between PNPLA3 abundance and other fixed effects (treatment and time) was evaluated through Spearman correlation; all correlations were r < 0.25 and deemed inconsequential. We report the slope (β) and *P*-value of liver PNPLA3 abundance from the mixed effect regression model and consider evidence of an association significant or marginal when *P* ≤ 0.05 or 0.05 < *P* ≤ 0.10, respectively. Additionally, we computed R^2^_V_ for the full mixed effect model, as well as the partial R^2^_V_ for the fixed effect of liver PNPLA3^23,68^.

#### Experiment 2

Data from the in vitro experiment, protein abundance and cellular TG, were expressed as the Log_2_ fold change of the experimental triplicate (siNON or siPNPLA3) relative to the basal treated cells. We elected to use this atypical transformation because initial model fittings exhibited non-homogenous variance across cell preparation (or calf) that was not rectified by standard data transformation (i.e. logarithmic, reciprocal, and power), inclusion of basal treated cell data as covariates, or modeling heterogenous variance. The LMM used to interrogate differences between the siRNA treatments included fixed effects of cell preparation and siRNA treatment, as well as accounting for heterogenous variance between cell preparations (except for ATGL protein abundance). Location tests of the least squares means (null hypothesis: mean = 0) were evaluated for each treatment to suggest effects of the transfection methodology on cell responses, relative to basal treated cells. Significant evidence for a difference between siRNA treatments was declared when *P* ≤ 0.05 and marginal evidence was declared when 0.05 < *P* ≤ 0.10.

## Supplementary Information

Supplementary Information.

## Data Availability

The data that support the findings of this study are available, on reasonable request, from the corresponding author.
