# Supplementary material for: Greater liver PNPLA3 protein abundance in vivo and in vitro supports lower triglyceride accumulation in dairy cows
Source: Sci Rep. 2021 Feb 2;11:2839. doi: 10.1038/s41598-021-82233-0 (PMC7854614; doi:10.1038/s41598-021-82233-0)
Supplement: Supplementary file 1 — Supplementary Information. [file 41598_2021_82233_MOESM1_ESM.docx]

**Greater liver PNPLA3 protein abundance *in vivo* and *in vitro* supports lower triglyceride accumulation in dairy cows**

**Ryan S. Pralle, Sophia J. Erb, Henry T. Holdorf, and Heather M. White^*^**

Department of Dairy Science, University of Wisconsin-Madison, Madison 53706

^*^Corresponding author: Heather M. White, Department of Dairy Science, University of Wisconsin-Madison, 1675 Observatory Drive, Rm 934B, Madison WI, 53706, Office: 608-263-7786, Fax: 608-263-7786, email: heather.white@wisc.edu

**SUPPLEMENTAL METHODS**

***Adipose Tissue Biopsy***

Subcutaneous adipose samples were collected from the tailhead region, sampling alternate sides of the tailhead over consecutive sampling dates. First, the tailhead region was shaved, including the sacral vertebrae, coccygeal vertebrae, and pins. The sacrococcygeal space was identified via palpation and an epidural (5 to 7 mL of lidocaine hydrochloride injectible-2%, Clipper Distribution Company, St. Joseph, MO) was given. When the cow tail became limp, the surgical field was prepared by alternating washes of povidone iodine (0.75% titratable iodine, First Priority, Elgin, IL) and 70% ethanol solutional, 3 each. Local anesthetic (10 mL of Lidocaine) was administered subcutaneously at the planned incision site. A straight-line incision was made (~3 cm) and a sterile 8 mm punch biopsy tool (Miltex model 33-57, Integra LifeSciences, Princeton, NJ) was used to collect adipose tissue (1 to 2 g). Then, the incision was sutured with a sterile non-absorbable suture material (USP 1, Braunamid white, Jorgensen Lab, Loveland, CO). The incision site and general health of the cow was monitored by the research staff and a veterinarian for 5 d post operation. Sutures were removed at 7 to 10 d post operation. After adipose tissue collection, the sample was immediately rinsed with saline, aliquoted into tubes (~500 mg per tube), flash frozen in liquid nitrogen, and stored at -80° C until further analysis.

***Hepatocyte Isolation***

Primary bovine hepatocytes were isolated from 3 Holstein bull calves less than 7 d old (average ± SD; 5 ± 2 d) with each individual calf represented an individual biological replicate. The caudate process was excised and perfused using collagenase at 37° C to maintain viability; hepatocytes were isolated from the caudate process as previously described^69,70^. Cells were plated on 35-mm tissue treated dishes (0030700112; Eppendorf, Hauppauge, NY) at a density of 2 million cells per plate in sterile Dulbecco’s Modified Eagle’s Medium (**DMEM;** D2902, low-glucose, no glutamate media; Sigma-Aldrich, St. Louis, MO) with added cell culture grade or higher 4-(2-hydroxyethyl)-1-piperazineethanesulfonic acid and sodium bicarbonate (H4034 and S5761; Sigma-Aldrich, St. Louis, MO) supplemented with 20% fetal bovine serum (F4135; Sigma-Aldrich, USA origin, Heat Inactivated, sterile-filtered, suitable for cell culture) and 1% antibiotic, antimycotic solution (A5955; Sigma-Aldrich, St. Louis, MO). Four h after initial plating, media was refreshed with sterile DMEM supplemented with 10% fetal bovine serum and 1% antibiotic, antimycotic. Cells were maintained in monolayer cultures for 24 h and were at least 80% confluent prior to treatment.

***Sample Protein Isolation***

***Experiment 1.*** Protein was isolated from 300 mg adipose tissue and 250 mg liver tissue by homogenization in a phosphate buffered saline lysis buffer with 1.0% nonidet P-40 substitute (US Biological, Salem, MA), 0.5% sodium deoxycholate (Sigma-Aldrich, St. Louis, MO), 0.1% sodium dodecyl sulfate (SDS; Sigma-Aldrich), and protease inhibitors (Halt^TM^ Protease and Phosphatase Inhibitor Cocktail; Thermo Scientific, Rockford, IL). Homogenates were centrifuged at 14,000 x *g* at 4° C for 5 minutes, after which the supernatant was collected for protein quantification. When obtaining the supernatant from tissue, the fat cake was avoided. A tissue specific pool of samples was created from equal volumes as a quality control standard for Western Blot analysis. Bicinchoninic assay (BCA; Pierce Biotechnology Inc., Rockford, IL) was used to determine protein concentration of all samples and pools via the manufacturer’s protocol. Samples originating from adipose tissue, but not liver tissue, were subjected to molecular weight concentrators of either 10 kDa or 30 kDa by following the manufacturer’s protocol (88513 and 88502, respectively; Pierce, ThermoFisher, Rockford, IL). After the concentration step, adipose tissue samples were re-analyzed via BCA assay to determine concentration. Samples originating from liver tissue, but not adipose tissue, were diluted and re-assayed as necessary. Final concentrations of adipose and liver tissues fell within the standard curve of each plate and the coefficient of variation of standards and samples never exceeded 10%.

***Experiment 2.*** Cells were harvested in 0.5 mL TRIzol reagent (Invitrogen, Carlsbad, CA) and stored at -80° C in RNA-free tubes for subsequent isolation via the manufacturer’s protocol (Life Technologies, ThermoFischer Scientific, Rockford, IL). Using a phenol-chloroform extraction method^59^, the aqueous phase was removed, and molecular grade ethanol was added to the remaining phenol-intermediate phases. Samples were centrifuged at 2,000 x *g* for 5 min at 4° C per the manufacturer’s protocol (15596018; Life Technologies), and phenol-ethanol fractions collected into new RNA-free tubes for protein isolation. Protein was precipitated with 100% isopropanol, centrifuged at 12,000 x *g* for 10 min at 4° C, washed with 0.3 M HCl buffer in 95% molecular grade ethanol, and centrifuged at 7,500 x *g* for 5 min at 4° C (Life Technologies). The supernatant was discarded, and the resulting protein pellet was re-suspended in a 10 mM EDTA, 140 mM NaCl, 100 mM Tris, 5% SDS lysis buffer adapted by previous optimization^71^ with protease inhibitors (Halt^TM^ Protease and Phosphatase Inhibitor Cocktail). Re-suspended protein pellets were then warmed for 30-45 min at 55° C, centrifuged at 10,000 x *g* for 10 min at 4° C, and supernatant collected across technical triplicates for downstream analysis. A pool of samples was created from equal volumes of supernatants collected as a quality control standard for Western Blot analysis. Samples were assayed via BCA assay as done in Experiment 1; all samples and pool fell within the standard curve and CV never exceeded 10%.

***Western Blotting***

Western Blotting was completed for both Experiment 1 and Experiment 2 following the same protocol and methodology as detailed here. A standardized protein quantity of 25 µg was used for pool and experimental sample preparation in Laemmili Buffer (161-0737; Bio-Rad Laboratories, Hercules, CA) and 0.2 M dithiothreitol (DTT25; Gold Biotechnologies, St. Louis, MO) then heated at 37° C for 30 min before gel loading. Prepared protein samples were loaded into a gradient gel of 4-20% Criterion TGX Stain-Free Protein Gels (Bio-Rad Laboratories) and proteins were separated by electrophoresis in Tris/Glycine/SDS Running Buffer (1610732; Bio-Rad Laboratories) for 40-45 min at 200 V. After electrophoresis, gels were activated on a ChemiDoc XRS+ (Bio-Rad Laboratories) for one minute and transferred to a polyvinylidene fluoride membrane utilizing the TransBlot Turbo transfer system using the “Midi, Mixed Molecular Weight” setting (Bio-Rad Laboratories). Membranes were blocked for 2 h at room temperature then incubated with a rabbit-derived primary antibody: PNPLA3 (ab81874; Abcam, Cambridge, MA) or adipose triglyceride lipase (ATGL; ab99532; Abcam). Primary PNPLA3 antibody incubation lasted 1 h at room temperature with dilutions of 1:250 and 1:500 for adipose and liver (tissue and hepatocytes) samples, respectively. Hepatocyte samples were incubated overnight at 4° C with the primary ATGL diluted at 1:3,000. Subsequently, blots were incubated with a goat-anti-rabbit secondary antibody for 1 h at room temperature diluted to 1:5,000 (ab97080 and ab99532 for PNPLA3 and ATGL, respectively; Abcam). After each antibody incubation, blots were washed with tris-buffered saline containing Tween for 4 times for 5 min each. Blots were imaged on the ChemiDoc XRS+ (Bio-Rad Laboratories) using ImageLab 5.0 software (Bio-Rad Laboratories) for 1) Total Lane Protein image using the Stain-Free setting and no imaging substrate and 2) bands of interest image using the ChemiHi Sensitivity setting with administration of SuperSignal West Dura Extended Duration Substrate at a 1:1 ratio (Pierce Biotechnology, Thermo Scientific, Rockford, IL). Probing order of PNPLA3 and ATGL on hepatocyte blots was done randomly; however, after images of the blot were taken for either ATGL or PNPLA3, blots were stripped with Restore Stripping Buffer (21059; ThermoFisher Scientific) for 20 min before re-blocking for 2 h at room temperature in order to analyze the alternate probe. Intra- and inter- assay CV of the pool on blots never exceed 15%.

***Experiment 1: Linear Mixed Model Fitting***

Initially, a base linear mixed model (LMM) model was fitted that incorporated the essential fixed and random effects for the response. The typical fixed effects included treatment, time, and treatment × time; the typical random effects included cow, block (expected calving week), cow nested within week of lactation (models with subsampling), and repeated measures of cow across time. After initial LMM fitting, responses were interrogated for the incorporation of covariates (i.e. previous lactation 305 d mature equivalent milk yield, parity, and -28 DRTC measurement), which were retained when there was sufficient evidence (*P* < 0.10) for a main effect or interaction with the treatment effect. Not all models had beneficial covariates, and no models had sufficient evidence (*P* < 0.10) for more than one beneficial covariate. Following covariate fitting, the conditional studentized residuals were subjectively evaluated by plotting (i.e. linear predictor × studentized residuals, studentized residual quantile-quantile plot, effect × studentized residuals). Homogeneity of variance between treatments was evaluated for all models via the likelihood ratio test employed by the GLIMMIX procedure and was retained when *P* < 0.05. When studentized residuals had a non-Gaussian distribution or unequal variance across linear predictors, modeling heterogeneous variance or alternative variance-covariance structures (default variance components) were investigated. Potential heterogeneous groups were determined based on plotting model variables by studentized residuals. Several heterogeneous groups and variance-covariance structures were investigated for each LMM; the reported LMM had the lowest Bayes information criteria or improved studentized residual plots. The final LMM for each response is detailed in Supplemental Table 4.

**Supplemental Table 1.** Least squares means (LSM) and standard errors of the mean (SEM) for tissue patatin-like phospholipase domain-containing protein 3 (PNPLA3) expression in their transformed scale used for linear mixed model (LMM) analysis across days relative to calving (DRTC) for cows exposed to a control (CTL) or ketosis induction protocol (KIP) treatment.

|  |  | Liver PNPLA3 mRNA^1^ | | | | | | |  | Liver PNPLA3 Protein^2^ | | | | | | |  | Adipose PNPLA3 Protein^3^ | | | | | | |
| --- | --- | --- | --- | --- | --- | --- | --- | --- | --- | --- | --- | --- | --- | --- | --- | --- | --- | --- | --- | --- | --- | --- | --- | --- |
|  |  | CTL | | |  | KIP | | |  | CTL | | |  | KIP | | |  | CTL | | |  | KIP | | |
| DRTC |  | LSM |  | SEM |  | LSM |  | SEM |  | LSM |  | SEM |  | LSM |  | SEM |  | LSM |  | SEM |  | LSM |  | SEM |
| - 14 |  | 2.21 |  | 0.36 |  | 1.18 |  | 0.33 |  | 2.05 |  | 0.03 |  | 2.01 |  | 0.04 |  | 2.17 |  | 0.08 |  | 2.13 |  | 0.08 |
| + 1 |  | 1.36 |  | 0.35 |  | 1.00 |  | 0.32 |  | 2.09 |  | 0.04 |  | 2.04 |  | 0.04 |  | 2.10 |  | 0.08 |  | 2.04 |  | 0.08 |
| +14 |  | 1.30 |  | 0.41 |  | 2.18 |  | 0.32 |  | 2.13 |  | 0.04 |  | 2.09 |  | 0.04 |  | 2.16 |  | 0.08 |  | 2.06 |  | 0.08 |
| +28 |  | 0.91 |  | 0.36 |  | 1.15 |  | 0.38 |  | 2.15 |  | 0.03 |  | 2.11 |  | 0.03 |  | — |  | — |  | — |  | — |
| +42 |  | 1.01 |  | 0.32 |  | 1.51 |  | 0.38 |  | 2.14 |  | 0.03 |  | 2.13 |  | 0.03 |  | — |  | — |  | — |  | — |
| +56 |  | 1.91 |  | 0.38 |  | 1.27 |  | 0.40 |  | 2.19 |  | 0.04 |  | 2.17 |  | 0.04 |  | 2.01 |  | 0.08 |  | 2.05 |  | 0.08 |

^1^Liver PNPLA3 mRNA expression values were originally expressed as PNPLA3, au relative to the geometric mean of reference gene expression, au. For LMM analysis, data were transformed: 1/x^0.5^.

^2^Liver PNPLA3 protein abundance values were originally expressed as PNPLA3, au relative to total lane protein × 100,000. For LMM analysis, data were transformed: Log_10_(x).

^3^Adipose PNPLA3 protein abundance values were originally expressed as PNPLA3, au relative to total lane protein × 10,000. For LMM analysis, data were transformed: Log_10_(x).

**Supplemental Table 2.** Spearman correlations (r) between -28 days relative to calving (DRTC) patatin-like phospholipase domain-containing protein 3 protein abundance and patatin-like phospholipase domain-containing protein 3 protein abundance at other timepoints within tissue.

|  |  | Liver | | |  | Adipose | | |
| --- | --- | --- | --- | --- | --- | --- | --- | --- |
| DRTC |  | r |  | *P*-value |  | r |  | *P*-value |
| - 14 |  | 0.77 |  | < 0.01 |  | 0.50 |  | 0.01 |
| + 1 |  | 0.63 |  | < 0.01 |  | 0.46 |  | 0.02 |
| +14 |  | 0.62 |  | < 0.01 |  | 0.55 |  | 0.03 |
| +28 |  | 0.71 |  | < 0.01 |  | – |  | – |
| +42 |  | 0.70 |  | < 0.01 |  | – |  | – |
| +56 |  | 0.64 |  | < 0.01 |  | 0.40 |  | 0.05 |

**Supplemental Table 3.** Partial Spearman correlations between tissue postpartum patatin-like phospholipase domain-containing protein (PNPLA3) expression, tissue metabolite concentrations, and energy balance.^1^

| Variable^2^ |  | lPNPLA3 mRNA, au |  | lPNPLA3 protein, au |  | aPNPLA3 protein, au |
| --- | --- | --- | --- | --- | --- | --- |
| lPNPLA3 mRNA, au |  | – |  | -0.04 |  | 0.15 |
| *P*-value |  | – |  | 0.78 |  | 0.34 |
| lPNPLA3 protein, au |  | -0.04 |  | – |  | -0.11 |
| *P*-value |  | 0.78 |  | – |  | 0.38 |
| aPNPLA3 protein, au |  | 0.15 |  | -0.11 |  | – |
| *P*-value |  | 0.34 |  | 0.38 |  | – |
| Liver TG, % DM |  | 0.10 |  | -0.32 |  | -0.03 |
| *P*-value |  | 0.55 |  | 0.01 |  | 0.84 |
| Serum BHB, mmol/L |  | -0.08 |  | 0.17 |  | 0.00 |
| *P*-value |  | 0.60 |  | 0.16 |  | 1.00 |
| Plasma glucose, mg/dL |  | 0.07 |  | -0.12 |  | 0.13 |
| *P*-value |  | 0.67 |  | 0.35 |  | 0.29 |
| Plasma FA, mEq/L |  | 0.06 |  | -0.39 |  | 0.08 |
| *P*-value |  | 0.70 |  | <0.01 |  | 0.52 |
| Energy balance, Mcal |  | -0.17 |  | 0.27 |  | 0.04 |
| *P*-value |  | 0.30 |  | 0.03 |  | 0.77 |

^1^Partial correlations controlled for the confounding effect of treatment

^2^Liver PNPLA3 = lPNPLA3, adipose tissue = aPNPLA3, TG = triglyceride, DM = dry matter, BHB = β-hydroxybutyrate, FA = fatty acid

**Supplemental Table 4.** Response variable transformation (ƒ(x)) and final linear mixed models for Experiment 1.

| Response | | | | |  | Model Parameters^4^ | | | | | | | | | | | | | | |
| --- | --- | --- | --- | --- | --- | --- | --- | --- | --- | --- | --- | --- | --- | --- | --- | --- | --- | --- | --- | --- |
| Variable^1^ |  | ƒ(x)^2^ |  | Stage^3^ |  | Covariate^5^ | | |  | TRT |  | Time |  | TxT |  | CxT |  | HV^6^ |  | VCOV^7^ |
| Body weight, kg |  | – |  | Study |  | -28 |  | <0.01 |  | 0.92 |  | <0.01 |  | 0.94 |  |  |  |  |  | VC |
| Body weight change, kg | | – |  | Pre |  | -28 |  | 0.05 |  | 0.93 |  | – |  | – |  |  |  |  |  |  |
|  |  | – |  | Post |  | -28 |  | <0.01 |  | 0.63 |  | – |  | – |  |  |  |  |  |  |
| BCS, points |  | 1/x |  | Study |  | -28 |  | <0.01 |  | 0.17 |  | <0.01 |  | 0.19 |  |  |  |  |  | VC |
| BCS change, points |  | – |  | Pre |  | -28 |  | <0.01 |  | 0.84 |  | – |  | – |  |  |  |  |  |  |
|  |  | – |  | Post |  | -28 |  | <0.01 |  | 0.03 |  | – |  | – |  |  |  |  |  |  |
| Milk yield, kg/day |  | x^2.25^ |  | Post |  | PME305 |  | <0.01 |  | 0.06 |  | <0.01 |  | 0.62 |  |  |  | WOL |  | VC |
| Milk protein yield, kg/day | | x^1.5^ |  | Post |  | – |  | – |  | 0.19 |  | <0.01 |  | 0.98 |  |  |  | WOL |  | VC |
| Milk fat yield. kg/day |  | x^1.25^ |  | Post |  | – |  | – |  | 0.96 |  | <0.01 |  | 0.08 |  |  |  | WOL |  | VC |
| Milk lactose yield, kg/day | | x^2.25^ |  | Post |  | PME305 |  | <0.01 |  | 0.17 |  | <0.01 |  | 0.77 |  |  |  | WOL |  | VC |
| Milk NEL, Mcal/day |  | x^2^ |  | Post |  | – |  | – |  | 0.65 |  | <0.01 |  | 0.10 |  |  |  | WOL |  | VC |
| Milk protein, % |  | 1/x^3^ |  | Post |  | – |  | – |  | 0.97 |  | <0.01 |  | 0.04 |  |  |  | TRT-Parity |  | VC |
| Milk fat, % |  | 1/x^1.25^ |  | Post |  | – |  | – |  | 0.25 |  | <0.01 |  | 0.81 |  |  |  | WOL |  | VC |
| Milk lactose, % |  | 1/x^1.5^ |  | Post |  | – |  | – |  | 0.19 |  | <0.01 |  | 0.53 |  |  |  | Block |  | VC |
| Milk solids not fat, % |  | – |  | Post |  | – |  | – |  | 0.52 |  | <0.01 |  | 0.16 |  |  |  | Block |  | VC |
| MUN, mg/dL |  | 1/x |  | Post |  | – |  | – |  | 0.92 |  | <0.01 |  | 0.78 |  |  |  | Parity |  | VC |
| SCC, cells/mL × 1,000 |  | 1/x^0.5^ |  | Post |  | – |  | – |  | 0.82 |  | <0.01 |  | 0.16 |  |  |  |  |  | VC |
| Milk NEL, Mcal/kg |  | 1/x |  | Post |  | – |  | – |  | 0.22 |  | <0.01 |  | 0.86 |  |  |  |  |  | VC |
| Dry matter intake, kg/day | | x^2.5^ |  | Pre |  | DPPD |  | <0.01 |  | 0.01 |  | <0.01 |  | 0.78 |  | 0.03 |  | TRT-Parity |  | VC |
|  |  | x^2.5^ |  | Post |  | DPPD |  | 0.02 |  | 0.05 |  | <0.01 |  | <0.01 |  |  |  | WOL |  | VC |
| Feed refused, % |  | x^0.5^ |  | Study |  | qFed |  | <0.01 |  | 0.02 |  | <0.01 |  | 0.08 |  |  |  |  |  | VC |
| NEL intake, Mcal/day |  | x^2.5^ |  | Pre |  | DPPD |  | <0.01 |  | <0.01 |  | <0.01 |  | 0.81 |  | 0.03 |  | TRT-WOL |  | VC |
|  |  | x^2^ |  | Post |  | DPPD |  | 0.02 |  | 0.05 |  | <0.01 |  | <0.01 |  |  |  | WOL |  | VC |
| Net energy balance, Mcal | | x^1.5^ |  | Pre |  | – |  | – |  | <0.01 |  | 0.04 |  | 0.27 |  |  |  | TRT |  | VC |
|  |  | – |  | Post |  | – |  | – |  | 0.39 |  | <0.01 |  | 0.04 |  |  |  | WOL |  | VC |
| Hepatic TG, % DM |  | Log_10_(x) | | Study |  | – |  | – |  | 0.41 |  | <0.01 |  | 0.76 |  |  |  |  |  | VC |
| lPNPLA3, au × 100,000 | | Log_10_(x) | | Study |  | -28 |  | <0.01 |  | 0.29 |  | <0.01 |  | 0.93 |  |  |  |  |  | ARH(1) |
| aPNPLA3, au × 10,000 | | Log_10_(x) | | Study |  | -28 |  | <0.01 |  | 0.56 |  | 0.35 |  | 0.75 |  |  |  |  |  | AR(1) |
| Plasma glucose, mg/dL |  | – |  | Study |  | – |  | – |  | 0.61 |  | <0.01 |  | 0.42 |  |  |  |  |  | ARH(1) |
| Serum BHB, mmol/L |  | 1/x |  | Study |  | – |  | – |  | 0.25 |  | <0.01 |  | 0.15 |  |  |  |  |  | ARH(1) |
| Plasma FA, mEq/mL |  | 1/x |  | Pre |  | – |  | – |  | 0.03 |  | <0.01 |  | 0.44 |  |  |  | Parity |  | VC |
|  |  | x^0.25^ |  | Post |  | Parity |  | <0.01 |  | 0.66 |  | <0.01 |  | 0.07 |  |  |  |  |  | VC |

^1^BCS = body condition score, NE_L_ = net energy of lactation, MUN = milk urea nitrogen, SCC = somatic cell count, TG = triglyceride, lPNPLA3 = liver patatin-like phospholipase domain-containing protein 3 (PNPLA3) protein abundance, aPNPLA3 = adipose PNPLA3 protein abundance, BHB = β-hydroxybutyrate, FA = fatty acid

^2^Mathmatical operations for data transformation of the response variable x to produce empirically (Shapiro-Wilk test) or subjectively (histogram) Gaussian distributions

^3^Timepoints included in the data analysis. Exp = the entire experimental period from -28 to +56 expected days relative to calving (DRTC), Pre = from -28 to 0 expected DRTC, Post = from +1 to +56 DRTC

^4^Statistics for fixed effects included in the respective models including the covariate, treatment (TRT), Time, the interaction of TRT and Time (T×T), and interaction of covariate and TRT (C×T). Additionally, information on the grouping factors for modeling heterogeneous variance (HV) and the selected variance-covariance (VCOV) for repeated measure models are provided.

^5^Selected covariates include the -28 DRTC sample value (-28), previous lactation 305 d mature equivalent milk production (PME305), days on prepartum experimental diet (DPPD),the quadratic effect of feed offered (qFed), and parity number.

^6^WOL = week of lactation, TRT-Parity = grouping based on treatment and parity number (*i.e.* control-parity 2, control-parity 4, and ketosis induction-parity 2)

^7^Variance covariance structures (VCOV) were selected based on their minimization of model Bayes information criterion. VC = variance components, AR(1) = first order autoregressive, ARH(1), heterogeneous first order autoregressive

**Supplemental Table 5.** Ingredient and nutrient composition of pre- and postpartum experimental diets.

| Diet Component |  | Prepartum^1^ |  | Postpartum^2^ |
| --- | --- | --- | --- | --- |
| Ingredient, % DM |  |  |  |  |
| Straw |  | 29.63 |  | – |
| Corn silage |  | 51.12 |  | 28.36 |
| Concentrate Mix^3^ |  | 19.25 |  | 36.07 |
| Alfalfa silage |  | – |  | 29.78 |
| Cottonseed |  | – |  | 5.80 |
| Chemical composition |  |  |  |  |
| DM, % |  | 43.70 |  | 48.71 |
| CP, % DM |  | 12.65 |  | 17.00 |
| NDF, % DM |  | 43.55 |  | 29.90 |
| Lignin, % DM |  | 4.62 |  | 4.18 |
| EE, % DM |  | 2.63 |  | 5.05 |
| NFC, % DM |  | 34.36 |  | 42.69 |
| Ash, % DM |  | 7.34 |  | 7.36 |
| NE_L_, Mcal/kg DM |  | 1.42 |  | 1.65 |

^1^Cows assigned to the ketosis induction protocol received 6 kg of dry cracked corn (90.2 % DM, 1.82 Mcal/kg DM) as a top-dress from -28 expected days relative to calving to parturition.

^2^Control and ketosis induction treatments received the same postpartum diet; however, ketosis induction cows were feed restricted to 80% of *ad libitum* intake from +14 days relative to calving until their blood β-hydroxybutyrate concentration was ≥ 3.0 mmol/L.

^3^Prepartum concentrate mix: Soybean meal 46 % CP (86.3 %), CaCO3 (3.37 %), Ca(H2PO4)2 (1.65 %), and a premix (8.68 %) composed of: CaSO4 (23.20 %), NaCl (13.85 %), CaCO3 (12.00 %), MgO (11.85 %), MgSO4 (11.80 %), CaHPO4 (21.0 %), mineral oil (1.0 %), selenium yeast 3000 (0.56 %, Prince Agri Products, Teaneck, NJ), Rumensin-90 (0.43%, Elanco Animal Health, Greenfield, IN), biotin (0.42%, DSM Nutritional Products, Belvidere, NJ), vitamin A (439.1 KIU/kg), vitamin D3 (132.7 KIU/kg), and vitamin E (6.4 KIU/kg). Postpartum concentrate mix: contained fine ground corn (55.78 %), canola meal (14.75 %), distillers grain (8.88 %), soy hull pellet (4.38 %), exceller meal 8.88 %, Quality Roasting Inc., Valders, WI), CaCO3 (2.25 %), NaHCO3 (2.25 %), grease (0.88 %), Urea (0.53 %), MgO (0.40 %), and a premix (1.05 %) composed of: Cl (51.2 %), Na (34 %), Ca (0.5 %), S (0.09 %), Co (78.2 ppm), Cu (4,871 ppm), I (469 ppm), Mn (14,382 ppm), Se (89.5 ppm), Zn (20,708 ppm), vitamin A (2055.7 KIU/kg), vitamin D3 (411.1 KIU/kg), vitamin E (8.7 KUI/kg), Rumensin-90 (1.8%, Elanco Animal Health, Greenfield, IN), and biotin (0.8%, DSM Nutritional Products, Belvidere, NJ).

**Supplemental Table 6.** Primer sequences used for quantitative real-time PCR.

| Gene |  | GenBank Accession |  | Position |  | Sequence |
| --- | --- | --- | --- | --- | --- | --- |
| *18S* |  | NR_036642.1 |  | Forward |  | 5'-ACCCATTCGAACGTCTGCCCTATT-3' |
|  |  |  |  | Reverse |  | 5'-TCCTTGGATGTGGTAGCCGTTTCT-3' |
| *RPL32* |  | NM_001034783.2 |  | Forward |  | 5'-AGACCCCTCGTGAAGCCTAA-3' |
|  |  |  |  | Reverse |  | 5'-CCGCCAGTTCCGCTTGATTT-3' |
| *PNPLA3* |  | XM_005207459.4 |  | Forward |  | 5'-ACGAAGGGTTCACCAAGCTC-3' |
|  |  |  |  | Reverse |  | 5'-GCATTAACAGCGACCGGAAC-3' |
